# Integrating ‘Omics’ Approaches to Prioritize New Pathogenetic Mechanisms for Mental Disorders

**DOI:** 10.1038/npp.2017.221

**Published:** 2017-12-01

**Authors:** Annamaria Cattaneo, Carmine M Pariante

**Affiliations:** 1 Stress, Psychiatry and Immunology Laboratory, Department of Psychological Medicine, Institute of Psychiatry, Psychology & Neuroscience, King’s College, London, UK; 2 Biological Psychiatry Unit, IRCCS Fatebenefratelli S. Giovanni di Dio, Brescia, Italy

Neuropsychopharmacology research is between a rock and a hard place. The rock is the historical, but slow, hypothesis-driven approach, where discovery occurs by testing candidate mechanisms in already well-known biological models. The hard place is the innovative, but overwhelming, hypothesis-free approach, where ‘omics’ analyses of everything that is analyzable generates a deluge of data implicating hitherto unknown mechanisms. So, either we have little data on things we already know, or too much data and cannot find the needle in a haystack. One solution is to mix apples and oranges: integrating cross-species and cross-tissues ‘omics’ data to find mechanisms that recur across different experimental and clinical models. The idea has been used with remarkable success. And yes, we will finish with the proverbs now.

Niculescu *et al* (2000) first developed and used such an approach, which they called convergent functional genomics. More recently, the approach has been used by them to help prioritize genes from genome-wide association studies (GWAS) of bipolar disorder (Patel *et al*, 2010), integrating GWAS findings, transcriptomics data on postmortem human brain and blood, and studies in animal models, to identify top-genes supported by all approaches. They identified six genes (*ARNTL*, *MBP*, *BDNF*, *NRG1*, *RORB*, and *DISC1*), which are involved in relevant biological processes, such as circadian rhythm, connectivity, and neuroplasticity. They used a similar strategy for schizophrenia (Ayalew *et al*, 2012). Interestingly, this strategy could be done with publically available data rather than being based on novel experimental findings.

In 2013, we studied transcriptomics data from the hippocampus of adult prenatally stressed rats (an established animal model of depression with high glucocorticoid levels) and from a human neuronal stem cell line (that we treated with a concentration of cortisol that reduces neurogenesis) (Anacker *et al*, 2013). We found that TGF*β*-SMAD2/3 and Hedgehog signaling are reduced in both models: TGF*β*-SMAD2/3 promotes neurogenesis (and has been found to be reduced in depressed patients), whereas Hedgehog promotes neuronal differentiation (and has not been studied in depressed patients yet). Similarly, Malki *et al* (2016) studied transcriptomics from the prefrontal cortex of mice bred for high aggressive behavior and from the brain of zebrafish exposed to aggressive social encounters. They identified seven genes shared in both datasets, including HDAC4, which has genetic variants associated with aggressive behavior in mental retardation, and it is targeted by valproic acid, a pharmacological treatment for aggressive behavior. Finally, Luoni *et al* (2016) studied methylome analyses performed in multiple models of early life stress: rats exposed to prenatal stress (prefrontal cortex); human newborns exposed to stress in pregnancy (cells from the umbilical cord); and rhesus monkeys exposed to stressful rearing conditions (peripheral blood and prefrontal cortex). Their top gene was *Ank3*, a gene with a strong association for psychiatric disorders; and they also demonstrated an interaction between functional genetic variants within *Ank3* gene and obstetric complications on working memory in humans. Although these studies are predominantly ‘comparative’ in their nature, this cross-species and cross-tissues approach can be used to produce ‘integrative’ findings when it generates novel lists of overlapping or functionally related genes through statistical or bioinformatic analysis.

With the collapse of R&D in mental health by pharmaceutical companies, convergent/integrative ‘omics’ approach represents a unique opportunity for the scientific community to mine existing datasets as well as data from experimental and clinical models, to prioritize targets for the psychotropic medications of the future.

## References

[bib1] Anacker C, Cattaneo A, Luoni A, Musaelyan K, Zunszain PA, Milanesi E et al (2013). Glucocorticoid-related molecular signaling pathways regulating hippocampal neurogenesis. Neuropsychopharmacology 38: 872–883. 23303060 10.1038/npp.2012.253 PMC3672002

[bib2] Ayalew M, Le-Niculescu H, Levey DF, Jain N, Changala B, Patel SD et al (2012). Convergent functional genomics of schizophrenia: from comprehensive understanding to genetic risk prediction. Mol Psychiatry 17: 887–905. 22584867 10.1038/mp.2012.37 PMC3427857

[bib3] Luoni A, Massart R, Nieratschker V, Nemoda Z, Blasi G, Gilles M et al (2016). Ankyrin-3 as a molecular marker of early-life stress and vulnerability to psychiatric disorders. Transl Psychiatry 6: e943. 27824361 10.1038/tp.2016.211 PMC5314123

[bib4] Malki K, Du Rietz E, Crusio WE, Pain O, Paya-Cano J, Karadaghi RL et al (2016). Transcriptome analysis of genes and gene networks involved in aggressive behavior in mouse and zebrafish. Am J Med Genet B Neuropsychiatr Genet 171: 827–838. 27090961 10.1002/ajmg.b.32451

[bib5] Niculescu AB 3rd, Segal DS, Kuczenski R, Barrett T, Hauger RL, Kelsoe JR (2000). Identifying a series of candidate genes for mania and psychosis: a convergent functional genomics approach. Physiol Genomics 4: 83–91. 11074017 10.1152/physiolgenomics.2000.4.1.83

[bib6] Patel SD, Le-Niculescu H, Koller DL, Green SD, Lahiri DK, McMahon FJ et al (2010). Coming to grips with complex disorders: genetic risk prediction in bipolar disorder using panels of genes identified through convergent functional genomics. Am J Med Genet B Neuropsychiatr Genet 153B: 850–877. 20468069 10.1002/ajmg.b.31087

